# An Integrated Single-Nucleus Atlas Resolves Cell-Type-Specific Programs and Molecular Subtypes in Alzheimer’s Disease

**DOI:** 10.64898/2026.09.03.747935

**Published:** 2026-09-04

**Authors:** Negin Rahimzadeh, Samuel Morabito, Saniya Khullar, Zechuan Shi, Zhenkun Cao, Vivek Swarup

## Abstract

Interindividual heterogeneity in Alzheimer’s disease (AD) remains poorly understood, as disparate single-cell studies leave it unclear whether findings reflect shared architecture or dataset-specific idiosyncrasies. Here, we present panAD, a transcriptomic atlas of >3 million nuclei from 791 individuals across 13 studies, spanning AD, mild cognitive impairment, and cognitively normal aging. AD converges on a reproducible, cell-type-specific molecular architecture: co-expression modules track neuropathology and cognitive decline; GWAS risk genes act predominantly as downstream targets of transcription factor hubs such as microglial *SPI1* ; intercellular communication is remodeled with disease stage; and sex differences concentrate in microglial immune-activation programs. To model patient-level transcriptomic heterogeneity, we developed the Multi-seed Optimization of Neural Embeddings for subTyping (MONET) framework, in which a masked variational autoencoder applied to covariate-adjusted, multi-cell-type profiles resolves four subtypes (Metal-Ion Stress, Neuroinflammatory, Synaptic Integrity, and Tissue Remodeling) that dissociate neuropathological burden from cognitive impairment and nominate predominantly non-overlapping candidate therapeutics. Finally, Stellar Atlas provides an AI-native conversational interface to the atlas.

## Full Text Availability

The license terms selected by the author(s) for this preprint version do not permit archiving in PMC. The full text is available from the preprint server.

